# Action Without Awareness: Reaching to an Object You Do Not Remember Seeing

**DOI:** 10.1371/journal.pone.0003539

**Published:** 2008-10-27

**Authors:** Matthew Heath, Anika Maraj, Bryan Godbolt, Gordon Binsted

**Affiliations:** 1 School of Kinesiology, The University of Western Ontario, London, Ontario, Canada; 2 Department of Electrical and Computer Engineering, The University of Western Ontario, London, Ontario; 3 School of Human Kinetics, University of British Columbia, Kelowna, British Columbia, Canada; Harvard Medical School, United States of America

## Abstract

**Background:**

Previous work by our group has shown that the scaling of reach trajectories to target size is independent of obligatory awareness of that target property and that “action without awareness” can persist for up to 2000 ms of visual delay. In the present investigation we sought to determine if the ability to scale reaching trajectories to target size following a delay is related to the pre-computing of movement parameters during initial stimulus presentation or the maintenance of a sensory (i.e., visual) representation for on-demand response parameterization.

**Methodology/Principal Findings:**

Participants completed immediate or delayed (i.e., 2000 ms) perceptual reports and reaching responses to different sized targets under non-masked and masked target conditions. For the reaching task, the limb associated with a trial (i.e., left or right) was not specified until the time of response cuing: a manipulation that prevented participants from pre-computing the effector-related parameters of their response. In terms of the immediate and delayed perceptual tasks, target size was accurately reported during non-masked trials; however, for masked trials only a chance level of accuracy was observed. For the immediate and delayed reaching tasks, movement time as well as other temporal kinematic measures (e.g., times to peak acceleration, velocity and deceleration) increased in relation to decreasing target size across non-masked and masked trials.

**Conclusions/Significance:**

Our results demonstrate that speed-accuracy relations were observed regardless of whether participants were aware (i.e., non-masked trials) or unaware (i.e., masked trials) of target size. Moreover, the equivalent scaling of immediate and delayed reaches during masked trials indicates that a persistent sensory-based representation supports the unconscious and metrical scaling of memory-guided reaching.

## Introduction

Visual awareness of the physical properties of a to-be-touched or to-be-grasped target object does not limit effective interactions with that object. The most striking demonstration of this phenomenon is exemplified in individuals with action-blindsight: a deficit arising from lesions to the primary visual cortex (V1) [1], [ for reviews see 2]–[3]. Persons with action-blindsight report being “unaware” of visual stimuli within their impaired hemifield; however, such individuals demonstrate preserved saccades, visually guided pointing and tracking within their scotoma [see also 4]. A more subtle demonstration of action without awareness is also observed following lesions to the lateral occipitotemporal cortex (LOC) [5]. In particular, the extensive studies of DF demonstrate that although impaired in identifying object shape and orientation (i.e., visual agnosia), she is readily able to scale her actions to the metrical properties of a target [6], [for review see 7].

Not surprisingly, the clinical neuropsychology literature has spawned interest in whether or not “action without awareness” can be observed independent of a *chronic* visual processing deficit [for chronic transcortical impact of V1 lesions see 8]. For example, Ro's [9] work reports that transient V1 disruption via single-pulse transcranial magnetic stimulation impacts the perceptual identification of a remote distractor but does not diminish the extent to which the same distractor facilitates movement planning (i.e., the redundant target effect) [see 10]. Further, extensive work using a double-step paradigm demonstrates that automatic and online limb adjustments arising from a change in target location can occur in the absence of visual awareness [11]–[12]. As well, work by our group [13]–[14] has shown that visual awareness of an intrinsic target property (i.e., size) is not necessary for appropriate size-scaling of reach trajectories [15]. Taken together, the results described just above indicate that visual awareness is not a precursor to motor output and that action-blindsight is not a restrictive clinical deficit; rather, the phenomenon reflects a general visuomotor characteristic.

The neural basis for the separation between conscious visual awareness and motor output is provided by Goodale and Milner's perception/action model (PAM) [7]. The PAM states that V1 or extrageniculate projections to the posterior parietal cortex of the dorsal visual pathway mediate motor output whereas projections from V1 to the inferotemporal cortex of the ventral visual pathway mediate conscious visual judgments. Thus, visuomotor processes are retained in the face of clinical or experimental disruption to early visual processing areas (i.e., V1) because extrageniculate projections to dorsal visuomotor networks can proxy for V1 inputs. Additionally, visuomotor processes are not influenced by disruption to the ventral visual pathway because dorsal visuomotor networks are not dependent on a conscious (i.e., top-down derived) visual percept.

An interesting question related to dorsal visuomotor function is the timeframe by which unconscious and metrical information can be retained and used to support motor output (so-called memory-guided action). A strong view of the PAM asserts that dorsal visuomotor networks operate along an evanescent time frame; that is, unconscious and metrical information is available only on a moment-to-moment basis (real time processing) [16], [for review see 17]. As such, introducing even the briefest of delay (i.e., 0 ms) between the occlusion of a visual stimulus and the onset of a response is proposed to nullify metrical reaching and grasping. Support for this view is garnered from reports that visually guided - but not memory-guided - responses are refractory to the context-dependent properties of pictorial illusions [18]–[21]. According to the real time position of the PAM, such results demonstrate that in the absence of continuous visual contact with a target object, memory-guided responses are mediated by a context-dependent visual percept laid down and maintained by the ventral visual pathway. However, the degree to which pictorial illusions provide a systematic and reliable means to address the timeframe of dorsal visual processing is questioned by accumulating evidence that illusions “trick” both visually and memory-guided responses [22]–[25; for reviews of this issue see 26–27].

An alternative to real time processing holds that visuomotor networks maintain a spatially enriched [28] and temporally durable representation [29]. Consistent with this assertion, recent work by our group [14] has employed a variant of Di Lollo et al's [30] four-dot object-substitution masking paradigm to demonstrate visuomotor memory in the absence of a conscious record [13]. In our previous work, participants were asked to verbally report the size of a target circle or reach (with their right hand) to that same target circle under conditions wherein the target was primed (i.e., no-mask trials) or perceptually masked (i.e., mask trials). Importantly, reaches were cued concurrent with presentation of the target circle (i.e., immediate cuing) or following a visual delay of 1000 or 2000 ms. Consistent with previous implementations of this masking procedure, perceptual reports of target size were correctly identified during no-mask (mean accuracy of verbal report = 88%, d' = 1.66) but not mask trials (mean accuracy of verbal report = 54%, d' = 0.17). Most importantly however, for the reaching task, movement times and other representative kinematic measures scaled to veridical target size [15] independent of mask condition (i.e., no-mask and mask) and across the immediate and delay (1000 and 2000 ms) conditions. Put another way, the absence of visual awareness did not preclude the veridical scaling of reach trajectories for up to 2000 ms of visual delay.

A question arising from our previous work relates to how (and where) unconscious visual information is used to support the sensory-to-motor transformations underlying metrical memory-guided reaching performance. One scenario holds that a movement plan related to target size is pre-computed at stimulus presentation (via parieto-frontal networks) and stored in memory to support later motor output [31]. An alternative scenario holds that visual target information is retained by dorsal visuomotor networks and subsequently accessed for conversion into a motor plan at - and not before - response cuing [32]. Thus, the present investigation sought to determine if visual information for which we are not aware is immediately used to specify the kinematic parameters of a memory-guided response or whether such information is retained in sensory form and used to support response specification at the time of movement cuing. In order to test these accounts, we again employed the four-dot masking paradigm to manipulate participant's perceptual awareness of target size [13] and included the maximal delay condition (i.e., 2000 ms) used previously [14]. Importantly however, the hand performing the response was not specified until response cuing; unimanual left and right hand responses were randomly interleaved on a trial-by-trial basis (i.e., two distinct auditory imperative tones designated left and right limb performance). We reasoned that specifying the limb only at the time of response cuing would preclude advanced sensory-to-motor transformations. Thus, if a pre-computed motor plan supports memory-guided reaches to a perceptually masked target, then precluding limb specification during the 2000 ms delay condition should nullify the metrical scaling of reach trajectories. In other words, precluding the specification of response parameters at initial stimulus presentation should render reach trajectories that are refractory to the size differences of a perceptually masked target. In contrast, if dorsal visuomotor networks retain a visual target representation, then such information should be flexibly able to support delayed motor output.

## Methods

### Participants

Thirty individuals from the University of Western Ontario community participated in this experiment. Participants were right handed [33] and had normal or corrected-to-normal vision. Participants gave written informed consent for a protocol approved by the University of Western Ontario's Office of Research Ethics (Review #14041S), and this work was conducted in accord with the 1964 Declaration of Helsinki.

### Apparatus and Procedure

Participants sat at a custom-built three-shelved aiming apparatus for the duration of this experiment [for pictorial depiction see 34]. The top shelf supported an inverted computer monitor (30-inch Monitor, 14 ms response time; 60 Hz: Dell 3007WFP: Round Rock, TX, USA), the middle shelf was composed of a one-way mirror (96 cm wide by 65 cm deep), and the bottom shelf was composed of a solid surface (96 cm wide by 65 cm deep). The distance between each shelf was 34 cm; thus, the optical geometry of our setup created a situation in which stimuli projected onto the mirror appeared to participants as being located on the bottom shelf (i.e., the reaching surface) of the aiming apparatus. In addition, head position was restrained via a head-chin rest (ASL-6000: Bedford, MA, USA). The reaching surface contained a home position defined by a haptic cue (5×3×3 cm magnet) located at participant's midline and 10 cm from the front edge of the apparatus. Because the one-way mirror occluded direct limb vision, dual light emitting diodes (LEDs) were placed at the home position to allow for the pre-movement visual calibration of limb position [35]. Computer events and all visual and auditory stimuli were controlled via MatLab (7.6: The MathWorks, Natick, MA, USA) and the PsychToolBox (ver. 3.0) [36].

**Figure 1:**
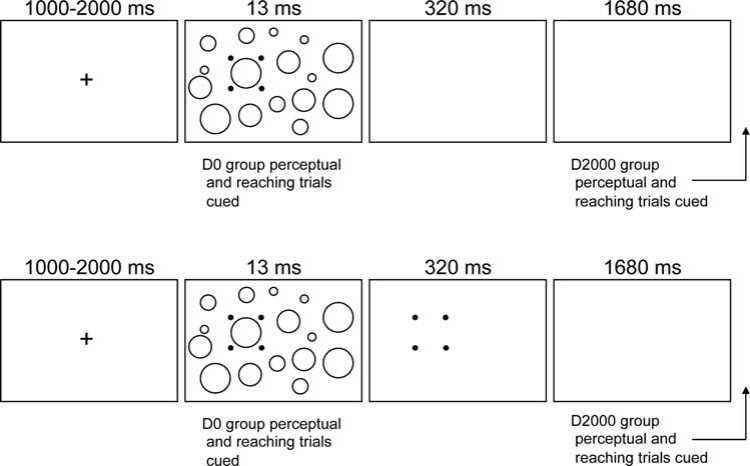
Schematic of the sequence of visual and auditory events associated with the performance of no-mask (top panels) and mask (bottom panels) trials for D0 and D2000 groups. Actual trials presented the four-dot mask as solid red circles; in this schematic the four-dot mask is simply represented by the filled circles (see panel two). For the D2000 group, perceptual and reaching responses were cued following offset of the fourth panel in the timeline (i.e., 2000 ms after onset of panel two). Note that due to scaling limitations only four circles of each target size are shown in the stimulus array.

Participants were presented with a central fixation cross (1 cm by 1 cm) for a randomized foreperiod (1000–2000 ms) after which an array of differently sized circles (1, 2, 3, and 4 cm) was presented for 13 ms (an array contained 5 randomly placed circles of each size). Within the array, one circle served as the “target” and was identified by four small red dots (i.e., the four-dot mask) arranged in an imaginary square (16 cm^2^) that surrounded but did not touch the target. Notably, the size of the four-dot mask was constant across all experimental trials. In the no-mask condition, the circles array and four-dot mask were extinguished simultaneously (i.e., after the 13 ms presentation) and replaced with a blank screen. In the mask condition, the four-dot mask remained visible for 320 ms after occlusion of the circles array (see Figure 1 for timeline of experimental events). Target circles were located 26.5 cm anterior to the home position and 8.5 cm left (i.e., left space) and right (i.e., right space) of participant's midline.

All participants completed perceptual and reaching trials in separate and counterbalanced trial blocks. Half of the participants (n = 15) were cued to complete their perceptual and reaching trials in time with onset of the circles array (0 s delay; i.e., the D0 group). The remaining participants completed their perceptual and reaching trials 2000 ms following onset of the circles array (i.e., the D2000 group).

#### Perceptual Task

To avoid confusion with the naming of intermediate-sized circles [see also 13]–[14], only the 2 and 4 cm circles were used as targets. These circles were identified in advance of the perceptual block and were labeled as “small” and “large” respectively. For each trial, participants were prompted (via auditory tone) to report (forced-choice binary decision) whether the target was small or large. The prompt was provided immediately (i.e., the D0 group) or 2000 ms (i.e., the D2000 group) after onset of the circles array. No-mask and mask trials were completed in separate and randomly ordered blocks. Within no-mask and mask blocks, small and large targets were randomly presented in left and right space on five occasions for a total of 20 perceptual trials.

#### Reaching Task

Participants were instructed to place their left and right index fingers on the home position in advance of each trial. At this position the fingers were spaced by approximately 2 cm. The goal of the reaching task was to point to the cued target as “quickly and accurately” as possible in response to an auditory tone. Because a trial could involve the performance of the left or the right hand, 300 Hz and 950 Hz, 13 ms tones were used to identify left and right hand performance respectively. Seven familiarization trials for each hand-tone combination were provided in advance of the reaching task. For the D0 group, the initiation tone was provided concurrent with onset of the circles array (see panel 2 of Figure 1). For the D2000 group, the initiation tone was provided 2000 ms after onset of the circles array (following panel 4 of Figure 1). Target sizes included 1, 2, 3 and 4 cm (i.e., each circle size within the array was used as a target) and produced respective index of difficulty (ID) values of 3.7, 4.1, 4.7 and 5.7 bits [log_2_(2A/W] (where A = amplitude and W = target width) [15]. In line with the perceptual task, targets circles were located 26.5 cm anterior to the home position and 8.5 cm left and right of midline (i.e., resultant movement vector of 278 mm). As such, participants reached to targets in ipsilateral (e.g., left hand-left target) or contralateral (e.g., left hand-right target) space. No-mask and mask trials were completed in separate blocks and within each block hand (left vs. right hand), target location (left space vs. right space) and target ID (3.7, 4.1, 4.7 and 5.7 bits) were randomly ordered with five trials completed to each combination for a total of 160 reaching trials.

Infrared emitting diodes (IRED) were attached to the nail of the left and right index fingers. IRED position data were sampled for 1.5 s at 200 Hz via an OPTOTRAK Certus (Northern Digitial Inc., Waterloo, ON, Canada). Offline, IRED position data were filtered at 10 Hz via a second-order dual-pass Butterworth filter. Instantaneous velocities and accelerations were computed via a three-point central finite difference algorithm. Movement onset was defined as the first frame that exceeded 50 mm/s for ten consecutive frames (50 ms) and movement offset was the first frame falling below 50 mm/s for ten consecutive frames.

### Dependent Variables and Statistical Analysis

For the perceptual task, the percentage of correct responses in no-mask and mask trials was examined via 2 (group: D0, D2000) by 2 (stimulus presentation: no-mask, mask) mixed-design ANOVA. For the reaching task, we computed reaction time (RT), movement time (MT), times to resultant peak acceleration (tPA), velocity (tPV) and deceleration (tPD) and resultant error (RE). Dependent variables for the reaching task were examined via 2 (group: D0, D2000) by 2 (stimulus presentation: non-mask, mask) 2 (hand: left hand, right hand) by 4 (target ID: 3.7, 4.1, 4.7 and 5.7 bits) mixed-design ANOVA. Only significant effects are reported and we report regression equations and R^2^ values as a means for interpreting significant effects/interactions. Means and between-participant standard deviations are reported in parentheses. Note that to streamline our results section, and in line with earlier work [13], [14], we did not include target location (i.e., left versus right space) in our ANOVA model. It is, however, important to note that RTs and MTs for left and right hand responses in ipsilateral space were faster than contralateral space, F(1,28) = 5.16, and 283.07 respectively for RT and MT, ps<0.05, and ipsilateral responses were always more accurate than contralateral ones, F(1,28) = 87.28, p<0.001. Importantly, target location did not interact with stimulus presentation or group (Fs<1.0).

## Results

### Perceptual Task

Target size was judged more accurately during no-mask (85% SD11, d' = 1.80 SD0.94) than mask trials (58% SD12, d' = 0.31 SD0.47), F(1,28) = 124.55, p<0.001. It is also noteworthy to mention that group and stimulus presentation did not interact (F<1.01).

### Reaching Task

**Figure 2:**
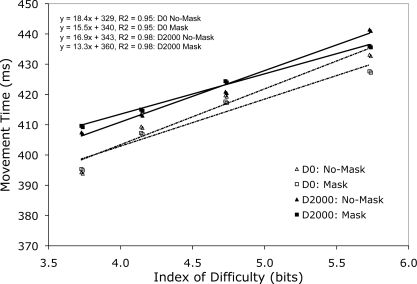
Movement time (ms) for D0 and D2000 group no-mask and mask trials as a function of target index of difficulty. In addition, the top left hand corner of the figure presents regression equations and R^2^ for each experimental condition.

Examination of RT yielded an interaction involving group and stimulus presentation, F(1,28) = 4.49, p<0.05. D0 group RTs during no-mask trials (438 ms SD89) were slower than mask trials (408 ms SD100) (t(14) = 2.62, p<0.03); however, D2000 group RTs were comparable across no-mask (412 ms SD81) and mask (409 ms SD69) trials (t(14) = 0.44, p>0.05). For MT, left hand reaches (429 ms SD80) were slower than right hand ones (404 ms SD76), F(1,28) = 41.04, p<0.001, and MT scaled in relation to increasing target ID, F(3,84) = 7.42, p<0.001. As shown in Figure 2 (see also Table 1), regression equations and R^2^ values relating movement time to target ID across D0 and D2000 group mask and no-mask trials indicate a reliable and robust increase in movement time as a function of increasing target ID. Moreover, examination of Figure 2 indicates that MT elicited null effects for stimulus presentation by target ID, as well as group by stimulus presentation by target ID (Fs<1.3).

**Table 1:** Reaction time (RT: ms), movement time (MT: ms), time to peak acceleration (tPA: ms), time to peak velocity (tPV: ms), time to peak deceleration (tPD: ms) and resultant error (RE: mm) as a function of target index of difficulty.

Dependent Variable	Index of Difficulty (*bits*)				Regression Equation	R^2^
	3.7	4.1	4.7	5.7		
RT	416 (91)	422 (86)	419 (86)	414 (75)	y = 420−0.9x	0.11
MT	401 (76)	411 (78)	420(77)	434(79)	y = 390+10.8x	0.97
tPA	110 (69)	112(75)	114 (80)	120 (79)	y = 106+3.2x	0.91
tPV	270 (64)	276 (66)	281 (69)	290 (67)	y = 263+6.5x	0.98
tPD	353 (72)	359 (70)	368 (71)	374 (71)	y = 345−7.2x	0.99
RE	6.2 (25)	4.8 (24)	5.0 (25)	4.7 (25)	y = 272+0.39x	0.57

In addition, regression equations and R^2^ values for each dependent variable are depicted.

Values are means. Between-participant standard deviations are presented in parentheses.

The time to achieve peak acceleration, velocity and deceleration for the left hand (tPA = 124 ms SD68, tPV = 290 ms SD59, tPD = 376 ms SD71) was longer than the right hand (tPA = 103 ms SD87, tPV = 268 ms SD67, tPD = 352 ms SD69), Fs(1,28) = 6.79, 23.90, and 32.14 respectively for tPA, tPV and tPD, ps<0.02. In addition, each measure increased with increasing target ID, Fs(3,84) = 5.30, 7.97, and 5.65 respectively for tPA, tPV and tPD, ps<0.03. As demonstrated in Table 1, regression equations and R^2^ values indicate that the impact of target ID on tPA, tPV and tPD was such that increasing target ID resulted in an increase in the time to achieve each kinematic marker. Last, analysis of RE indicated that mask trials (1.5 mm SD28.7) were more accurate than no-mask trials (8.8 mm SD20.6), F(1,28) = 6.10, p<0.03.

## Discussion

The goal of this investigation was to determine how visual information that is unavailable to conscious verbal report is used to support the scaling of memory-guided reaching. In particular, we sought to determine if unconscious and metrical information related to an intrinsic target property (i.e., size) is used to pre-compute the parameters of a memory-guided response or whether such information is maintained as a sensory (i.e., visual) representation for on-demand sensorimotor conversion at response cuing. To that end, participants completed immediate (i.e., D0) or memory-based (i.e., D2000) verbal reports and reaching (left and right hand) responses to perceptually masked targets using a variant of Di Lollo et al's [30] four-dot masking paradigm [see also 13]. Importantly, a critical response parameter associated with the reaching task (i.e., the limb performing the movement) was specified only at the time of response cuing thereby limiting the pre-computing of an advanced motor plan.

### Re-entrant processing and the perceptual masking of target size

In line with previous work [13]–[14], [Bibr pone.0003539-DiLollo1] [30; for review see 37], verbal reports during no-mask trials achieved a robust level of accuracy whereas mask trials operated at chance. According to Di Lollo et al's [30] computational model of object substitution, the simultaneous offset of target and non-target items during non-mask trials allows for uniform decay of visual features and permits a stable visual percept to be laid down and accessed by high-level visuo-perceptual networks (i.e., the ventral visual pathway). In contrast, the asymmetric offset of target and non-target items during mask trials elicits non-uniformity of decay; that is, re-entrant processing of non-target features at low-level visual processing areas (i.e., V1) conflicts with a “visible persistence” of target features maintained by high-level visual processing areas. As such, re-entrant processing renders the original percept (i.e., target and non-target features) unavailable for conscious report. It is also worthy to note that in our study the D0 and D2000 groups showed equivalent performance across no-mask and mask trials. In particular, the equivalent findings for no-mask trials across the two groups used here indicates that when consciously perceived, the visuo-perceptual networks of the ventral visual pathway maintain a temporally durable representation of target size [7].

### Four-dot masking and the scaling of reaching trajectories

Before addressing the principal issue of how perceptual masking and motor uncertainty impact the size-scaling of memory-guided reaching, we outline the general impact of our limb manipulation. First, specifying the limb at response cuing (i.e., left or right hand) resulted in longer reaction times (Grand Mean = 416 ms) than a similar experiment employing only right hand reaches (Grand Mean = 234 ms) [see 14]. Of course, the between-experiment difference represents an expected result owing to the increased stimulus response alternatives used here [38]–[39]. Moreover, the longer planning times evidence that motor parameters were not pre-computed at the time of stimulus presentation; rather, the reaction times shown here indicate that selection of the limb performing the response in combination with specifying the movement parameters for that limb occurred in time – and not before - response cuing [32]. Second, the use of left and right hand responses yielded an expected asymmetry in response execution such that the right hand elicited faster movement times and achieved representative kinematic markers sooner than left hand counterparts [for review of this issue see 40].

We did not find that reaction time was sensitive to target ID and this null effect generalized across mask and no-mask trials for D0 and D2000 groups. In other words, results provide no evidence that reaction time scaled in relation to target size [cf. 13], [41]. We did, however, observe that D0 group no-mask trials exhibited slower reaction times than mask trial counterparts whereas D2000 group reaction times did not vary across no-mask and mask trials. Recall that D0 group no-mask trials involved the simultaneous blanking of the visual array and onset of the auditory imperative tone whereas in the other experimental conditions the imperative tone was provided in time with persistence of the four-dot mask (i.e., D0 group mask trials) or after all elements of the visual array were extinguished (i.e., D2000 group mask and no-mask trials) (see Figure 1 for timeline of experimental events). It is therefore possible that the double stimulus cue provided during D0 group no-mask trials delayed movement planning processes [cf. 42].

Although reaction time did not scale to target ID, Figure 2 shows that movement times for D0 and D2000 groups increased as a function of increasing target ID for both mask and no-mask trials. Figure 2 also demonstrates equivalent slopes relating movement time to target ID across the different experimental conditions. Moreover, the times to achieve peak acceleration, peak velocity and peak deceleration for D0 and D2000 group no-mask and mask trials demonstrated a scaling effect with target ID. Thus, results from our experiment demonstrate that across all conditions lawful speed-accuracy trade-offs related to target size emerged during the response evocation stage of reaching. As noted by a myriad of studies, this effect is taken to reflect the need to devote longer movement durations to ensure that a response “hits” the desired target location [15]; [for review of this issue see 43]. It is also worth mentioning that our study did not provide participants with online limb vision: a manipulation quite different from Fitts' original work [15], [41] wherein participants were afforded continuous limb vision. Indeed, the fact that we observed speed-accuracy relations on par to that reported by Fitts indicates that speed-accuracy relations are not entirely determined by feedback-based limb corrections. Rather, our results are in line with accumulating evidence that speed-accuracy relations are in part determined by central planning mechanisms [for discussion of this issue see 27].

The combined results of the perceptual and reaching task match previous work by our group and indicate that awareness of target size is not necessary to support the metrical scaling of immediate or memory-guided reaches involving up to 2000 ms of delay [13]–[14]. Moreover, the current investigation adds importantly to the extant literature in demonstrating that unconscious and metrical information supporting memory-guided reaches reflects a sensory (i.e., visual) representation maintained by dorsal visuomotor networks. The basis for this assertion is predicated on the fact that our limb manipulation – and introduction of premovement motor uncertainty - prevented participants from pre-computing the kinematic parameters of their reach trajectories in advance of response cuing. In particular, the limb associated with any given trial for the D2000 group was specified well after extinction of the target object. Thus, the ability of the D2000 group to scale their reach trajectories to veridical target size mandated that a sensory representation be maintained in memory until the time of response cuing.

In general, the present results support the PAM's assertion that dorsal visuomotor networks operate independent of an obligatory visual percept [7]. However, the present results are inconsistent with the PAM's contention that dorsal visuomotor networks operate along an evanescent timeframe (i.e., real-time control) [see 16]. As mentioned in the Introduction, the real time nature of dorsal visuomotor function is supported by some work involving memory-guided reaching/grasping of pictorial illusions [20; but see 26]–[27 for alternative views] and the studies of patient DF (i.e., visual agnosia) demonstrating a breakdown in her ability to scale reach and grasp trajectories following a memory delay [6]. In a complementary manner, there exists some data involving an individual (i.e., GY) with action-blindsight to report null scaling between grip aperture and target size when a delay is introduced between target presentation and the onset of a movement within the impaired hemifield [44]. It is, however, important to note that Weiskrantz et al's [1] classic study of DB demonstrates preserved visuomotor function in the presence of a visual delay. In particular, Weiskrantz et al. presented a static visual target for a 2000 ms preview and the extinction of the target served as the experimenter's cue to verbally prompt DB to initiate his reaching response. Of course, the time required for the experimenter to perceive offset of the visual target and the time for the experimenter to produce the verbal imperative in combination with the time required for DB to plan and initiate his response would have introduced an appreciable period of visual delay (>1000 ms). Thus, and although we are unable to offer specific insight into the nature of the discrepant literature provided above, we believe that findings from a clinical patient [1] as well as the present and other work by our group [13]–[14] provides convergent evidence that unconscious and metrical visual information is retained as a sensory based representation and is available to support visuomotor processes for up to 2000 ms of visual delay. Indeed, future work is set to provide a systematic probe of the impact of increasing memory delays (i.e., immediate reaching, 0, 500, 1000, 1500, and 2000 ms of delay) on movement scaling in persons with documented action-blindsight and matched controls [45]. The goal of this future work is to ascertain whether the persistence of unconscious and metrical information in the aforementioned groups is susceptible to differing decay properties.

A final issue requiring redress relates to the impact of our experimental manipulations on endpoint accuracy. Similar to previous work [13], target ID did not influence the accuracy of reaching responses. That finding in combination with the temporal measures described above indicates that emergent speed-accuracy relations were defined by the timing, and not the spatial, properties of the movement goal. It was also observed that mask trials were more accurate than no-mask trials. In line with our previous work [13]–[14] we attribute such a finding to the improved ocular gaze anchoring [46] and spatial landmarking [47] afforded by the four-dot mask. More specifically, the four-dot mask provided additional spatial information allowing for more effective target localization.

### Conclusions

Here we demonstrate that the scaling of memory-guided reaching movements to target size is not dependent on an obligatory visual percept. Moreover, by precluding the specification of a movement parameter during the delay interval used here, we establish that a persistent sensory (i.e., visual) representation supports the unconscious and metrical scaling of memory-based actions. Such findings indicate that the visuomotor networks of the dorsal visual pathway retain a spatially enriched and temporally durable sensory-based representation that is distinct from that subserving perception based activities.
